# Leucine-Rich Glioma Inactivated 1 Promotes Oligodendrocyte Differentiation and Myelination via TSC-mTOR Signaling

**DOI:** 10.3389/fnmol.2018.00231

**Published:** 2018-07-06

**Authors:** Ya-Jun Xie, Lin Zhou, Yin Wang, Nan-Wei Jiang, Shenglong Cao, Chong-Yu Shao, Xin-Tai Wang, Xiang-Yao Li, Ying Shen, Liang Zhou

**Affiliations:** ^1^Key Laboratory of Medical Neurobiology of Ministry of Health, Department of Neurobiology, Zhejiang University School of Medicine Hangzhou, China; ^2^Laboratory of Craniocerebral Diseases of Ningxia Hui Autonomous Region, Ningxia Medical University Yinchuan, China; ^3^Ningbo Key Laboratory of Behavioral Neuroscience, Department of Physiology and Pharmacology, Ningbo University School of Medicine Ningbo, China; ^4^Department of Neurosurgery, Second Affiliated Hospital of Zhejiang University School of Medicine Hangzhou, China

**Keywords:** Lgi1, myelination, oligodendrocyte, oligodendrocyte precursor cell, mTOR

## Abstract

Leucine-rich glioma inactivated 1 (Lgi1), a putative tumor suppressor, is tightly associated with autosomal dominant lateral temporal lobe epilepsy (ADLTE). It has been shown that Lgi1 regulates the myelination of Schwann cells in the peripheral nervous system (PNS). However, the function and underlying mechanisms for Lgi1 regulation of oligodendrocyte differentiation and myelination in the central nervous system (CNS) remain elusive. In addition, whether Lgi1 is required for myelin maintenance is unknown. Here, we show that Lgi1 is necessary and sufficient for the differentiation of oligodendrocyte precursor cells and is also required for the maintenance of myelinated fibers. The hypomyelination in *Lgi1*^−/−^ mice attributes to the inhibition of the biosynthesis of lipids and proteins in oligodendrocytes (OLs). Moreover, we found that Lgi1 deficiency leads to a decrease in expression of tuberous sclerosis complex 1 (TSC1) and activates mammalian target of rapamycin signaling. Together, the present work establishes that Lgi1 is a regulator of oligodendrocyte development and myelination in CNS.

## Introduction

Leucine-rich glioma inactivated 1 (Lgi1), containing a leucine-rich repeat (LRR) domain, encodes a secreted protein in the central nervous system (CNS; Gu et al., 2002). Prevailing evidence demonstrates that Lgi1 is tightly associated with autosomal dominant lateral temporal lobe epilepsy (ADLTE; Senechal et al., 2005; Sirerol-Piquer et al., 2006; Head et al., 2007). Further studies indicate that Lgi1 is critical to the pruning of glutamatergic synapses in the hippocampus (Zhou et al., 2009) through the interaction with ADAM (a disintegrin and metalloproteinase; Fukata et al., 2006; Chabrol et al., 2010; Thomas et al., 2010). Consequently, the deletion or mutation in Lgi1 impairs glutamatergic transmission in hippocampus, which is considered to be the pathologic basis for ADLTE (Fukata et al., 2010; Yu et al., 2010).

Apart from epileptogenesis, it has been shown that Lgi1 plays roles in neuronal and glial development. The ablation of Lgi1 causes a subtle neuronal dyslamination in the cortex (Silva et al., 2015) and reduces the thickness of external granule cell layer in embryonic cerebellum (CB; Su et al., 2015; Xie et al., 2015). Members of Lgi family are yet involved in the myelination of Schwann cells in the peripheral nervous system (PNS). In particular, Lgi4 binds to ADAM22 and facilitates cellular interaction and axon sorting in the developing PNS (Özkaynak et al., 2010; Kegel et al., 2014) and the loss function of Lgi4 contributes to the abnormalities in claw paw (clp) mutant mice (Bermingham et al., 2006). Moreover, EM observations ON the sciatic nerve demonstrate Lgi1 knockout impairs myelination of axons in PNS (Silva et al., 2010). These findings raise a question whether Lgi1 coordinates the myelination of oligodendrocytes (OLs) in CNS.

In the present work, we show that Lgi1 is necessary and sufficient to the differentiation of oligodendrocyte precursor cells (OPCs) and myelination and is also required for the maintenance of myelinated fibers facing cuprizone challenge. The hypomyelination caused by Lgi1 deficiency attributes to inhibited biosynthesis of lipids and proteins in OLs. We further show that Lgi1 deficiency decreases the expression of tuberous sclerosis complex 1 (TSC1) and breaks the balance of mTOR signaling in OLs, which might be the cause of hypomyelination.

## Materials and Methods

### Animals

All experiments were approved by the Animal Experimentation Ethics Committee of Zhejiang University and were specifically designed to minimize the number of animals used. Original breeding pairs of *Lgi1*^−/−^ mice were obtained from John Cowell (Augusta University; Yu et al., 2010; Zhou et al., 2018) and were maintained at the Experimental Animal Center of Zhejiang University. Mice were kept under temperature-controlled condition on a 12:12 h light/dark cycle with food and water *ad libitum*. *In vivo* experiments were done in a batch of mice of either sex.

### Antibodies and Reagents

Antibodies against Olig2, CC1, MBP, myelin oligodendrocyte glycoprotein (MOG), 2′,3′-cyclic nucleotide 3′-phosphodiesterase (CNP), myelin associated glycoprotein (MAG), and GAPDH were purchased from Millipore. Antibodies to pS6 (S240/244), S6 and TSC1 were from Cell Signaling Technology. The antibody to platelet-derived growth factor α receptor (PDGFαR) was from Santa Cruz. Ki67 was from Abcam. Triiodothyronine (T3) and PDGF-AA were from Sigma. Horseradish peroxidase-conjugated secondary antibodies for immunoblotting were from GE Healthcare. IgG antibody, Dulbecco’s modified Eagle’s medium (DMEM), 4′,6-diamidino-2-phenylindole (DAPI), Alexa Fluor-conjugated secondary antibodies, neurobasal and B27 supplements were from Invitrogen. FBS was from GIBCO. Other chemicals were from Sigma unless stated otherwise.

### OPC Culture

Purified OPCs from SD rats were isolated by shaking off as described previously (Zhou et al., 2014). In brief, OPCs were collected from glial cultures by shaking for 1 h at 200 rpm, incubating in fresh medium for 4 h, and shaking at 250 rpm at 37°C for 16 h. Collected OPCs in the medium were re-plated onto poly-D-lysine-coated plates and grew in Neurobasal medium supplemented with 2% B27. PDGF-AA (10 nM) was added in the medium to keep OPCs undifferentiated. Alternatively, T3 (40 ng/ml) was added to the medium for 3 days to allow their differentiation (Li et al., 2013; Zhou et al., 2014).

### Lentivirus Construction and Transfection

Lentivirus encoding small hairpin RNA (shRNA) for Lgi1 (sequence: 5’-CCT AAG AGG GAA CTC ATT T-3’) was prepared by OBIO (Shanghai). Overexpressing Lgi1 was based on the coding sequence of rat Lgi1 gene (GenBank accession number 145769). Lgi1-shRNA and scrambled RNA were driven by U6 promoter, whereas overexpressed Lgi1 was driven by CMV promoter. OPCs were transfected with *Lgi1*-shRNA for 72 h and Lgi1 overexpression virus for 24 h before experiments. Only when >60% of cultured OPCs were transfected, which was confirmed by GFP fluorescence, experiments were continued.

### Western Blot

Proteins derived from tissues or cultured cells were rinsed with phosphate-buffered saline (PBS) and diluted in 1% SDS containing protease inhibitor cocktail. Protein concentration was determined using the BCA protein assay (Bio-Rad). Equal quantities of proteins were loaded and fractionated on sodium dodecyl sulfate-polyacrylamide gels (SDS-PAGE) and transferred to PVDF membrane (Immobilon-P, Millipore), immunoblotted with antibodies, and visualized by enhanced chemiluminescence (Pierce Biotechnology). Primary antibody dilutions used were Lgi1 (1:1000), MBP (1:10000), MAG (1:2000), MOG (1:2000), CNP (1:10000), pS6 (1:1000), S6 (1:1000), TSC1 (1:1000), fatty acid synthase (FASN; 1:500), and GAPDH (1:10000). Film signals were digitally scanned and quantitated using ImageJ 1.42q (NIH).

### Immunohistochemistry and Immunocytochemistry

Thirty micrometer sagittal sections were prepared and placed in blocking solution (1% BSA, 0.3% Triton, and 10% goat serum) for 1 h at room temperature (RT). After washing with PBS, sections were incubated sequentially with primary antibodies overnight at 4°C and secondary antibodies for 1 h at RT. The secondary antibodies were diluted at 1:1000. The sections were mounted using ProLong Gold Antifade Reagent with DAPI (Invitrogen). Cultured cells were fixed with 4% paraformaldehyde for 15 min at RT, washed with PBS and permeabilized in 0.2% Triton X-100 for 10 min, blocked in 10% BSA for 1 h, and labeled with primary antibodies overnight at 4°C, then cells were incubated with secondary antibodies (1:1000) for 1 h at RT. All antibodies were diluted in PBS containing 1% BSA and 1% normal goat serum. The dilution ratios of primary antibodies for immunohistochemistry and immunocytochemistry were MBP (1:1000), pS6 (1:1000), and FASN (1:100). To calculate the areas of cultured cells, somata and processes were manually tracked by tracing their edges. A process was determined by criteria including the explicit focus, the clear origin point from the soma, and the separation from other processes.

### Cell Counts

Three to four animals per genotype were used to examine the cellular marker expression for each time point. In the cortex, three to five nonadjacent sections were counted per animal. In the corpus callosum (CC), images were acquired to include only the CC at the midline.

### Eriochrome Cyanine Staining

Cryosections on glass slides were rinsed with PBS and submerged in Eri-C solution for 1 h. The sections were then differentiated in 10% (w/v) iron alum for 30 min until the nuclei became transparent. Slides were then dehydrated and coverslipped. All operations were performed at RT.

### Electron Microscopy (EM)

Tissues for the electron microscopy (EM) were prepared as described previously (Pereira et al., 2010; Zou et al., 2011). Ultra-thin sections were obtained using Ultracut UCT (Leica) and stained with 2% uranyl acetate and lead citrate. Electron micrographs were taken with a Philips CM100 microscope (FEI). The ratio of axonal diameter/fiber diameter (*g*-ratio) was acquired using ImageJ software.

### Human Material

The human study was approved by the Medical Ethical Committee of Zhejiang University School of Medicine and was conducted in conformance with policies and principles included in the Federal Policy for Protection of Human Subjects and in the Declaration of Helsinki. Informed written consents were given by all participants. Basic clinic information of these patients is given in Table 1. Brain tissue specimens containing neocortex were obtained as part of planned surgical margin of resection surrounding the tumor core. The para-cancerous tissue, tumor infiltration area and tumor mass area of glioma foci was distinguished by visible appearance and separated under an inverted microscope (Pallud et al., 2014). Aliquots of tissues from each patient were used for protein extraction and western blot, in which the subdivision of each area was confirmed by the different expression of PSD95 and GFAP (Louis et al., 2007).

**Table 1:** Clinic information.

# Patient	Gender	Age on surgery	Location of tumor	Affected hemisphere	WHO histological grade
1	Male	48	Frontal lobe	Left	Glioma IV
2	Female	48	Insular lobe	Right	Glioma III
3	Female	48	Frontal lobe	Right	Glioma IV
4	Male	42	Parietal lobe	Left	Glioma IV
5	Male	52	Frontal lobe	Right	Glioma IV
6	Female	54	Frontal lobe	Left	Glioma IV

### Statistics

Data analysis was performed using Excel 2003 (Microsoft), Igor Pro 6.0 (Wavemetrics), and SPSS 16.0 statistical program (SPSS). Statistical differences were determined using unpaired two-sided Student’s *t*-test. The accepted level of significance was *p* < 0.05. “*n*” represents the number of animals or cultures tested. Data in the text and figures are presented as mean ± SEM. The experimental protocols, analytic methods and study material that support this study are available from the corresponding authors upon reasonable request.

## Results

### Expressions of Lgi1 and MBP Are Correlated in OLs

**Figure 1:**
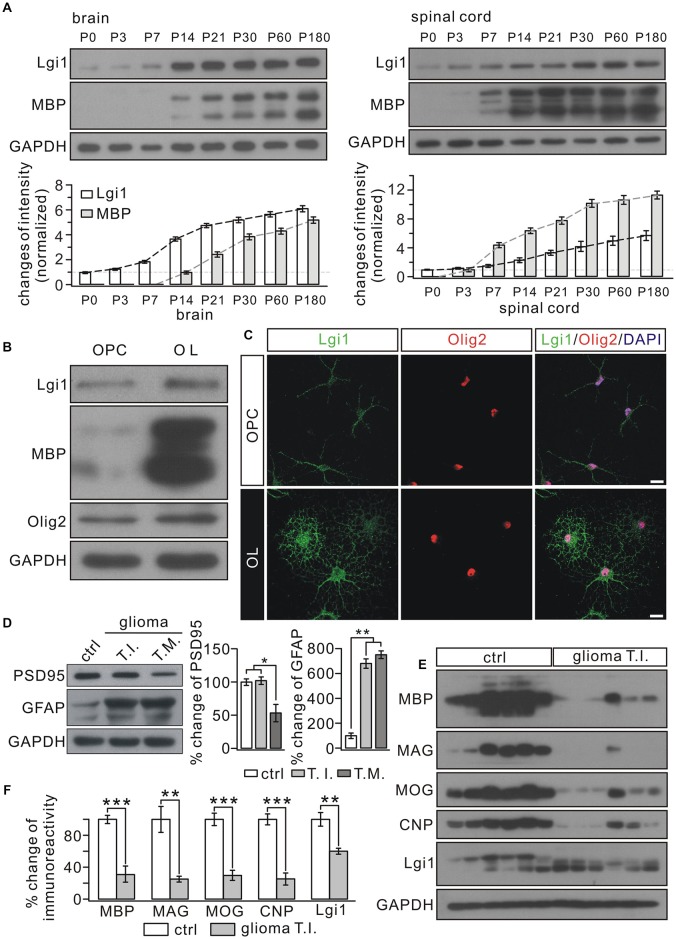
Correlated expression between Leucine-rich glioma inactivated 1 (Lgi1) and MBP. **(A)** The expressions of Lgi1 and MBP in the brain and spinal cord (SC) from postnatal mice. **(B)** Lgi1 expression was significantly enhanced in of oligodendrocytes (OLs) compared with in oligodendrocyte precursorcells (OPCs). **(C)** The immunocytochemical staining of Lgi1 and Olig2 in OPCs and OLs. Scale bars, 20 μm. **(D)** An example showing the expression of PSD95 and GFAP in para-cancerous tissue (ctrl), tumor infiltration (TI), and tumor mass (TM) from one patient. The experiment was repeated for 18 times as the samples were from six patients. Histogram shows percentage changes of PSD95 and GFAP relative to GAPDH, the loading control. *p < 0.05, **p < 0.01. **(E)** Western blots of protein expression in isolated tumor infiltration and corresponding control tissues from six patients. Each band represents a sample from one patient. The experiment was repeated for three times. **(F)** Bar graphs show the percentage changes of MBP, myelin associated glycoprotein (MAG), myelin oligodendrocyte glycoprotein (MOG), cyclic nucleotide 3′-phosphodiesterase (CNP) and Lgi1 after normalized to ctrl. MBP, ***p < 0.001. MAG, **p = 0.0048. MOG, ***p < 0.001. CNP, ***p < 0.001. Lgi1, **p = 0.0096.

We measured the expression of MBP and Lgi1 in the postnatal brain and spinal cord (SC). Western blots showed that MBP expression increased in proportion to Lgi1 in both regions (Figure 1A). Similarly, in cultured OPCs and mature OLs, we found that the expression of Lgi1 was significantly elevated in OLs compared with OPCs (Figure 1B). Immunocytochemical staining corroborated this result by showing high fluorescence of Lgi1 in OLs, which was not limited to soma but widespread in elaborated processes, a characterization of mature OL (Figure 1C). In order to further determine the correlation between Lgi1 and myelin proteins, we measured their expressions in glioblastomas. Total 18 fresh brain tissue specimens obtained from six patients (three tissues from each patient) with supratentorial, hemispheric, and diffuse high-grade gliomas (Table 1) were physically subdivided into control (*n* = 18), tumor infiltration (*n* = 18), and tumor mass (*n* = 18), which were confirmed by levels of GFAP and PSD95 in western blot (Figure 1D). In consistent with previous report (Besleaga et al., 2003), our results showed Lgi1 expression was significantly attenuated in gliomas (Figures 1E,F). Meanwhile, the expressions of myelin-associated proteins (MBP, MOG, MAG and CNP) were unanimously reduced in gliomas (Figures 1E,F). Hence, these results provide evidence showing the correlated expression between Lgi1 and myelin proteins.

### Lgi1 Deficiency Causes Hypomyelination in CNS

**Figure 2:**
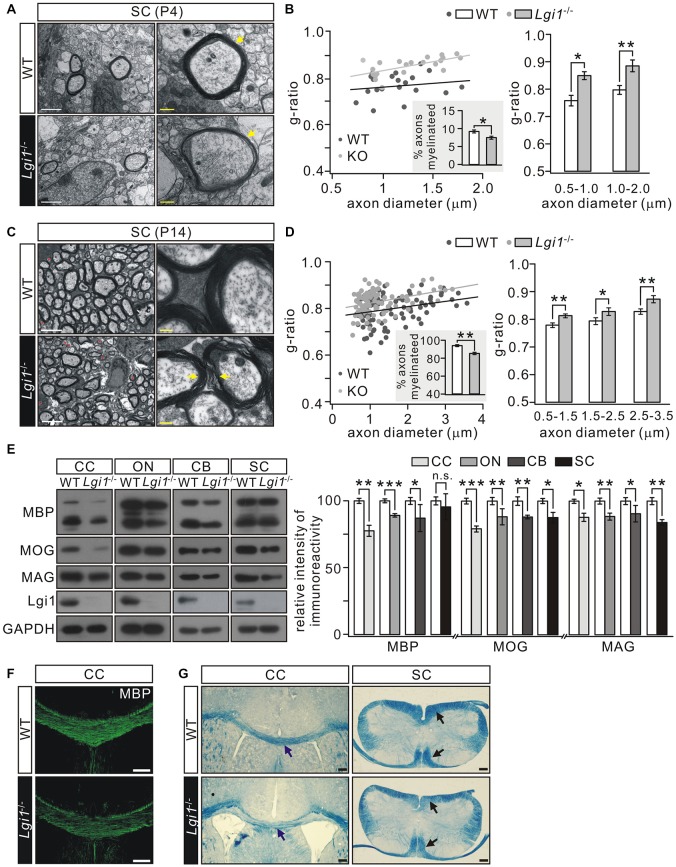
Deletion of Lgi1 reduces central nervous system (CNS) myelination. **(A)** Electron microscopies (EMs) from SC of wild-type (WT) and *Lgi1*^−/−^ mice (P4). Yellow arrows show thin and loose myelin sheath in *Lgi1*^−/−^ littermates. Scale bars, 0.5 μm (left panels) and 0.1 μm (right panels). **(B)** Average *g*-ratios (*n* = 4): 0.77 ± 0.02 (WT) and 0.83 ± 0.02 (*Lgi1*^−/−^) for axons of 0.5–1.0-μm diameters; 0.79 ± 0.02 (WT) and 0.86 ± 0.02 (*Lgi1*^−/−^) for axons of 1.0–2.0-μm diameters. The inset shows percentages of myelinated axons in P4 WT and *Lgi1*^−/−^ mice (*n* = 4). **(C)** EM studies in SC from WT and *Lgi1*^−/−^ mice at P14. Note that the organization of myelin was apparently loose in *Lgi1*^−/−^ mice, as indicated by yellow arrowheads. Scale bars, 0.5 μm (left panel) and 0.1 μm (right panel). Unmyelinated axons are indicated by red asterisks. **(D)** Average *g*-ratios (*n* = 4): 0.77 ± 0.01 (WT) and 0.80 ± 0.01 (*Lgi1*^−/−^) for axons with 0.5–1.5 μm diameters; 0.79 ± 0.01 (WT) and 0.82 ± 0.01 (*Lgi1*^−/−^) for axons of 1.5–2.5-μm diameters; 0.81 ± 0.01 (WT) and 0.86 ± 0.01 (*Lgi1*^−/−^) for axons of 2.5–3.5-μm diameters. The inset shows percentages of myelinated axons in P14 WT and *Lgi1*^−/−^ mice (*n* = 4). **(E)** Western blot of myelin protein expression in corpus callosum (CC), optic nerve (ON), cerebellum (CB), and SC from WT and KO mice (P14). CTX: *n* = 4. ON: *n* = 4. CB: *n* = 4. SC: *n* = 4. **(F)** MBP staining reveals a dramatic reduction of MBP-positive fibers in CC of *Lgi1*^−/−^ mice at P14. Scale bars, 100 μm. **(G)** Cyanine staining shows white matter tracts in the CC and SC from WT and *Lgi1*^−/−^ mice (P14), as indicated by arrowheads. Scale bars, 100 μm. *p < 0.05, **p < 0.01, ***p < 0.001, ns: no significance.

We next used EM to compare myelin formation in the SC from wild-type (WT) and *Lgi1*^−/−^ littermates. Several abnormalities were found in *Lgi1*^−/−^ mice at P4: (i) there appeared to be an increased incidence of loose myelin layers (Figure 2A); (ii) myelin thickness was reduced, which was revealed by morphometric quantification showing an increased *g*-ratio in myelin sheath of both smaller (0.5–1.0 μm) and larger (1.0–2.0 μm) diameters in *Lgi1*^−/−^ littermates (Figure 2B); and (iii) a reduction in the number of myelinated axons in *Lgi1*^−/−^ littermates (Figure 2B). These results suggest a hypomyelination phenotype in postnatal *Lgi1*^−/−^ mice. Moreover, this hypomyelination persisted in P14 *Lgi1*^−/−^ mice, as shown by increased *g*-ratio and loose myelin layers (Figures 2C,D) and a significant reduction in the number of myelinated axons compared to WT (Figure 2D). The hypomyelination in *Lgi1*^−/−^ mice is confirmed by western blot and immunohistochemistry. First, the expression of myelin proteins, including MBP, MOG and MAG, was prominently attenuated in the CC, ON, CB and SC from *Lgi1*^−/−^ mice at P14 (Figure 2E). Second, the immunostaining presented a broad loss of MBP-positive fibers in *Lgi1*^−/−^ mice (Figure 2F). Third, eriochrome cyanine staining showed that *Lgi1*^−/−^ mice had a significant reduction in the density of white matter tracts in both CC and SC of P14 *Lgi1*^−/−^ mice (Figure 2G). Taken together, these findings suggest that deletion of Lgi1 leads to myelination defects in CNS.

### Lgi1 Ablation Impairs OPC Differentiation in the Brain

**Figure 3:**
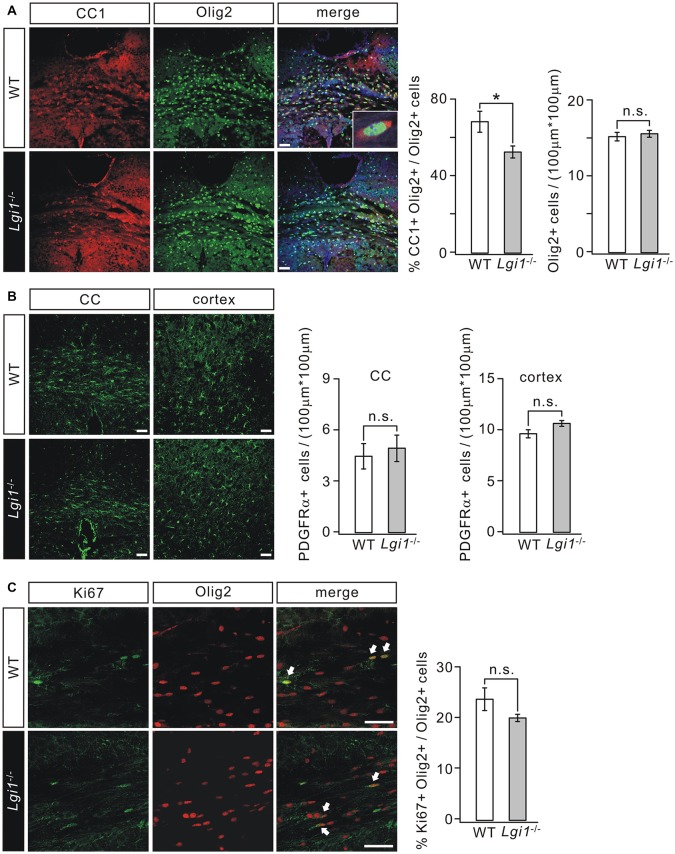
Deletion of Lgi1 impairs the differentiation of OPCs. **(A)** Immunostaining of differentiated OLs (CC1+/Olig2+ cells; inset) in CC of P14 WT and KO mice. Scale bar, 50 μm. Bar graphs show the averages of total Olig2+ cells (*p* = 0.42; *n* = 4) and the percentage of CC1+ cells in Olig2+ cells (*p* = 0.026; *n* = 6). **(B)** Immunostaining of platelet-derived growth factor α receptor (PDGFαR) in the CC and cortex of P14 mice. Scale bars, 50 μm. Bar graphs show the averages of total PDGFαR+ cells in CC (*p* = 0.177; *n* = 4) and cortex (*p* = 0.1248; *n* = 6). **(C)** Immunostaining of proliferating OLs (Ki67+/Olig2+ cells; indicated by arrows) in the CC of P14 mice. Scale bars, 50 μm. The bar graph shows percentages of Ki67+/Olig2+ cells in total Olig2+ cells (*p* = 0.1565, *n* = 4). *p < 0.05, ns, no significance.

To determine how Lgi1 deficiency causes hypomyelination, we analyzed the expression of cellular markers selected for OPC and OL. Our results showed that the total number of OLs indicated by Olig2+ cells in the CC from P14 *Lgi1*^−/−^ mice was not affected (Figure 3A). However, the number of differentiated OLs expressing CC1 and Olig2 simultaneously was reduced by 23% compared with WT (Figure 3A). These results suggest that Lgi1 contributes to OPC differentiation in the brain.

We continued to examine if the proliferating capacity of OPCs was influenced by Lgi1 ablation. PDGFαR staining in CC and cortex showed that there was no significant difference in the number of PDGFαR+ OPCs between WT and *Lgi1*^−/−^ littermates at P14 (Figure 3B). Furthermore, immunohistochemical staining with antibodies against Ki67 and Olig2 was used to assess the cell cycle of OPCs. Statistically, the number of proliferating OLs (Ki67+/Olig2+) of *Lgi1*^−/−^ mice was normal compared with that of WT in the CC (Figure 3C). These data suggest that Lgi1 is not required for OPC formation and proliferation.

### Lgi1 Is Necessary and Sufficient for OPC Differentiation

**Figure 4:**
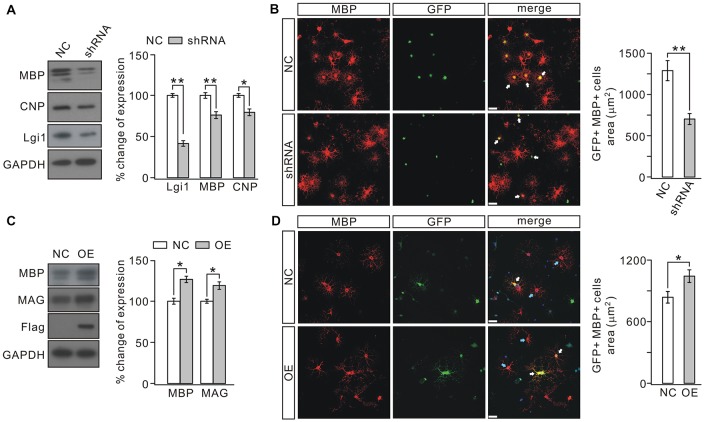
Knocking down or overexpressing Lgi1 affects the differentiation of OPCs. **(A)** Cultured OPCs were lentivirally transduced with scramble RNA (NC) or *Lgi1*-shRNA-GFP (shRNA). Western blots show the expression levels of MBP and CNP were decreased by shRNA (MBP, *p* = 0.0059; CNP, *p* = 0.0129; *n* = 4). As the control for lentivirus infection, Lgi1 expression was analyzed from same samples (*p* = 0.0037; *n* = 4). **(B)** Arrows present GFP+/MBP+ cells. Scale bars, 20 μm. Note the difference in the area and the development of secondary processes in NC and shRNA groups. The right panel shows the quantification of average areas of GFP+/MBP+ cells in NC or shRNA group (*p* = 0.0095, *n* = 4). **(C)** Cultured OPCs were transduced with control lentivirus (NC) or lentiviral plasmid encoding Lgi1 (OE). The levels of MBP and MAG proteins were increased by overexpressing Lgi1 (MBP, *p* = 0.022; MAG, *p* = 0.011; *n* = 4). Meanwhile, increased expression of Lgi1 was confirmed by the expression of Flag that was ligated to lentivirus. **(D)** White arrows indicate GFP+/MBP+ cells (upper and lower panels) and blue arrows indicate MBP+ cells (lower panel). The results show that the over-expression of Lgi1 (GFP+/MBP+ cells in lower panel) increases the area of OLs. Scale bars, 20 μm. The average areas of GFP+/MBP+ cells in NC or OE group (*p* = 0.0222, *n* = 4) was shown in the right panel. *p < 0.05, **p < 0.01.

*In vitro* observations were next used to determine the role of Lgi1 in OPC differentiation by changing its expression with lentiviral transfection in purified OPC cultures (Zhou et al., 2014). OPCs were transduced with GFP-tagged *Lgi1*-shRNA or scrambled RNA prior to T3 stimulation and affected cells were distinguished by GFP fluorescence. The efficiency of transfection was defined by western blot assay (Figure 4A). We found that T3 promoted the differentiation of OPCs in control group, as shown by characteristic extensive processes of MBP+ cells. In contrast, shRNA-induced *Lgi1* knockdown restricted the development of processes and remarkably reduced the area of MBP+ cells, though the number of MBP+ cells remained unchanged (Figure 4B). In consistent with immunocytochemical observations, the protein expression of MBP and CNP was also reduced in *Lgi1*-shRNA group (Figure 4A). These results indicate that Lgi1 may be necessary for OPC differentiation.

To investigate whether Lgi1 is sufficient to promote OPC differentiation, we transduced cultured OPCs with either control virus or lentiviral plasmid encoding Lgi1, as shown by the expression of Flag (Figure 4C). The OPC cultures were then treated with T3 for 1 day, a time shorter than normal OPC differentiation. Our results showed that overexpression of Lgi1 significantly increased the area of MBP+ cells compared with vector control (Figure 4D); and MBP and MAG were also elevated (Figure 4C), suggesting that Lgi1 overexpression enhances the differentiation capacity of OPC.

### Lgi1 Attenuates Cuprizone-Induced Myelin Degeneration

**Figure 5:**
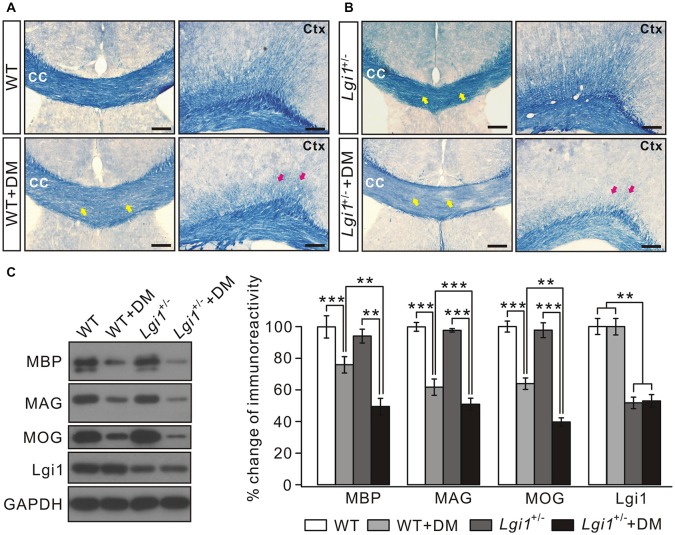
Cuprizone-induced loss of myelin sheath is severer in *Lgi1*^+/–^ mice. **(A)** Representative images of cyanine staining of CC and Ctx from 2-month WT mice. WT+DM: WT mice plus cuprizone diet. Yellow and red arrows indicate the reduction in white matter tracts in corpus CC and cortex. Scale bars, 100 μm. **(B)** Cyanine staining in the corpus CC from *Lgi1*^+/–^ mice (2 months). *Lgi1*^+/–^+DM: *Lgi1*^+/–^ mice plus cuprizone diet. Note that the loss of myelin sheath was much worse in *Lgi1*^+/–^ mice compared to WT. Scale bars, 100 μm. **(C)** The representative blots and quantification of MBP, MAG, MOG and Lgi1 expression in indicated situation (*n* = 4). WT+DM and WT: MBP, *p* < 0.001; MAG, *p* = 0.0042; MOG, *p* < 0.001; Lgi1, *p* = 0.56. *Lgi1*^+/–^+DM and *Lgi1*^+/–^: MBP, *p* = 0.0051; MAG, *p* < 0.001; MOG, *p* < 0.001; Lgi1, *p* = 0.48. *Lgi1*^+/–^+DM and WT+DM: MBP, *p* = 0.0031; MAG, *p* < 0.001; MOG, *p* = 0.0012; Lgi1, *p* < 0.001, **p < 0.01, ***p < 0.001.

The degeneration of myelin sheath occurs in many demyelinating diseases, for example multiple sclerosis (Raine et al., 1999) and in gliomas (Underhill et al., 2011; Liu et al., 2014; Mantero et al., 2015). It was of interest to explore whether Lgi1 is involved in myelin loss. To this end, we established a demyelination model by feeding 8-week old adult mice with a diet containing 0.2% cuprizone for 4 weeks. In this scenario, *Lgi1*^+/–^ mice was used because homozygous *Lgi1*^−/−^ mice died usually after P17 (Xie et al., 2015; Zhou et al., 2018). In agreement with previous work (Chen et al., 2012), cuprizone administration led to massive demyelination in cortical layers, cingulum bundles, and CC, as shown by greatly attenuated cyanine-stained myelin tracts in the CC and cortex (Figure 5A). Interestingly, cuprizone-induced loss of myelin sheath was much severer in *Lgi1*^+/–^ mice than WT (Figure 5B), indicating that the haploinsufficiency of Lgi1 exacerbates the degeneration of myelinated fibers. This conclusion was also confirmed by western blot, which showed the reduction in the expression of myelin-associated proteins, including MBP, MAG, and MOG, was more prominent in *Lgi1*^+/–^ mice compared with WT littermates when they were both treated with cuprizone (Figure 5C). Meanwhile, the myelin tracts (Figures 5A,B) and the expression of myelin proteins (Figure 5B) were not changed in *Lgi1*^+/–^ mice compared to WT mice, indicating that myelin maturation is not affected by the haploinsufficiency of *Lgi1*. Hence, these results demonstrate that Lgi1 prevents the myelin degeneration caused by cuprizone. However, the precise roles of Lgi1 in myelin maintenance shall be determined using adult mice with the deletion of Lgi1 by PLP-ER-Cre.

### Lgi1 Deficiency Inhibits Lipid Biosynthesis in OLs

**Figure 6:**
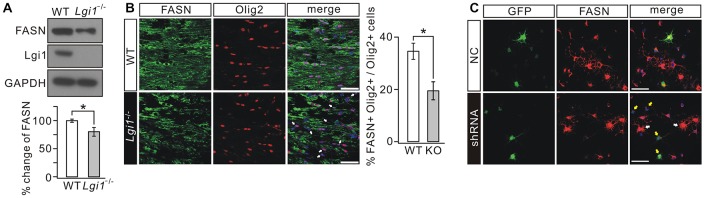
Deletion of Lgi1 reduces the expression of fatty acid synthase (FASN). **(A)** Cortical lysates from WT and *Lgi1*^−/−^ littermates at P14 were probed by immunoblotting with antibodies to FASN and Lgi1. FASN expression was significantly reduced in *Lgi1*^−/−^ mice compared to WT (*p* = 0.0297, *n* = 4). **(B)** Immunostaining of FASN and Olig2 in CC of P14 WT and *Lgi1*^−/−^ mice, as indicated by arrows. Scale bars, 50 μm. Bar graph shows the percentage of FASN+Olig2+ cells (*p = 0.0149; *n* = 4). **(C)** Cultured rat OPCs were lentivirally transduced with scramble RNA (NC) or *Lgi1*-shRNA-GFP (shRNA). FASN expression was much reduced in cells transduced with shRNA (yellow arrows) compared to cells that were not affected (white arrows). Scale bars, 100 μm.

Lipid contents provide the building blocks of multilayered membrane structure of OLs (Woelk and Borri, 1973; Fressinaud et al., 1990; Salles et al., 2002; Saher et al., 2005). Since increased *g*-ratio and loose myelin layers have been found in *Lgi1*^−/−^ mice (Figure 2), we investigated whether Lgi1 ablation changes the lipogenesis in OLs. Western blot revealed that the expression of FASN was significantly decreased in *Lgi1*^−/−^ mice (Figure 6A). Because the contribution from cell types other than OLs might have masked the attenuation of FASN, we performed immunohistochemistry on P14 CC. In *Lgi1*^−/−^ mice, Olig2+ OLs showed strongly diminished FASN staining present in the sections, leading to reduced FASN+Olig2+ cells in *Lgi1*^−/−^ mice (Figure 6B). This finding was corroborated and extended by *in vitro* experiments utilizing GFP-tagged *Lgi1*-shRNA in cultured OPCs, showing that the fluorescence of FASN was significantly reduced in shRNA-transfected cells compared with those unaffected (Figure 6C). These results suggest that Lgi1 ablation might impair myelin layers via altered lipid biogenesis in OLs.

### Lgi1 Deficiency Activates mTOR Signaling in OLs

**Figure 7:**
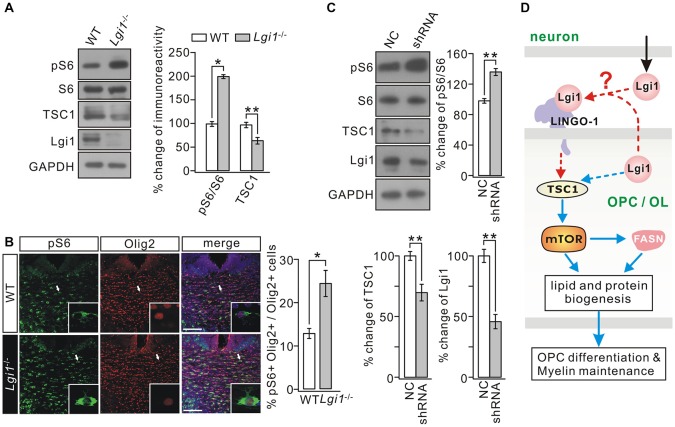
Deletion of Lgi1 activates mTORC1 signaling. **(A)** Western blot analysis of protein expression in isolated cortex of WT and *Lgi1*^−/−^ mice at P14. Right, quantification of protein levels (*n* = 3; pS6/S6, *p* = 0.0092; tuberous sclerosis complex 1 (TSC1), *p* = 0.0087; Lgi1, *p* = 0.0076). **(B)** Immunostaining of pS6 and Olig2 in the corpus CC of P14 WT and *Lgi1*^−/−^ mice, as shown by the white arrows and higher magnification. Scale bars, 50 μm. Bar graph shows the percentage of pS6+Olig2+ cells (*p = 0.0262; *n* = 3). **(C)** Cultured OPCs were lentivirally transduced with scramble RNA (NC) or *Lgi1*-shRNA-GFP (shRNA). Western blot analysis shows pS6/S6 was increased (**p = 0.0045) while TSC1 expression was decreased (**p = 0.0059) by shRNA (*n* = 3). Lgi1 expression from the same samples was the internal control for infection (**p = 0.0035; *n* = 3). **(D)** A schematic illustration shows proposed signaling pathways involved in the regulation of Lgi1 on OPC differentiation and myelin maintenance. Blue lines: intracellular pathway. Red lines: intercellular pathway.

The results above imply that Lgi1 deficiency changes intracellular signaling in OLs, but the downstream effectors of Lgi1 largely remain unknown. mTOR signaling is intimately linked with TSC1 (Laplante and Sabatini, 2009) and TSC1-mTOR signaling acts as a critical checkpoint for OL homoeostasis and proper CNS myelination (Norrmén and Suter, 2013; Lebrun-Julien et al., 2014; Zou et al., 2014; Jiang et al., 2016). Interestingly, mTOR regulates the lipogenesis in passage cell lines (Laplante and Sabatini, 2010; Soliman, 2011; Lamming and Sabatini, 2013) and in OLs (Lebrun-Julien et al., 2014). Accordingly, we investigated whether TSC1-mTOR signaling is altered and thereby underlies observed phenotypes in *Lgi1*^−/−^ mice. The expression of TSC1 and ribosomal protein S6, the substrate of mTORC1, and the phosphorylation of S6 (pS6) was examined in WT and *Lgi1*^−/−^ littermates. Indeed, we found that mTOR signaling is activated in *Lgi1*^−/−^ mice because pS6 was increased while TSC1 expression was decreased in these mice (Figure 7A). Given that this phenotype might hold partial contributions from other cell types, two experiments argued that TSC1-mTOR signaling was indeed altered in OLs. First, immunohistochemistry on CC sections demonstrated that the intensity of pS6 signal was strongly increased in Olig2+ OLs from *Lgi1*^−/−^ littermates compared to WT (P14) (Figure 7B). Second, pS6 was increased but TSC1 expression was reduced in shRNA-treated rat OPC cultures where Lgi1 expression was suppressed (Figure 7C). These results indicate that Lgi1 ablation upregulates mTORC1 pathway in OLs, which might further reduce myelin formation and cause demyelination.

## Discussion

Lig1 has been documented as an epilepsy-linked gene because Lgi1 mutations result in ADLTE (Kalachikov et al., 2002). Previous EM observations indicate that Lgi1 knockout impairs myelination in the PNS and CNS (Silva et al., 2010), however, the mechanisms underlying the function of Lgi1 in oligodendrocyte myelination remain elusive. Our present study demonstrates that Lgi1 promotes the differentiation of OPCs in part through activating the biosynthesis of lipids and proteins in OLs by tipping the balance of TSC1-mTOR signaling. We further find that Lgi1 is critical for the maintenance of myelinated fibers.

We observed that Lgi1 expression was positively correlated with MBP expression (Figure 1A) and Lgi1 was significantly elevated during OPC differentiation (Figures 1B,C). Moreover, the difference of *g*-ratio between WT and *Lgi1*^−/−^ mice became smaller at P14 compared to P4 (Figures 2A–D). These data suggest that there may be stage-dependent functions of Lgi1 in oligodendrocyte development, which needs to be confirmed by using conditional Lgi1-knockout mouse, as the global deletion of Lgi1 causes the death of animal (Xie et al., 2015) Importantly, the knockdown of Lgi1 in OPCs impaired OPC differentiation and maturation (Figure 4). The present work using isolated OPC hypothesizes a cell-intrinsic function of Lgi1 in OLs development and CNS myelination (Figure 7D, blue lines), distinct from Lgi4-induced reciprocal cell-neuron communications in PNS myelination (Özkaynak et al., 2010; Kegel et al., 2014). Nonetheless, we could not exclude the possibility of non-cell autonomous effects of Lgi1 loss in other neural cell types on myelinogenesis, because either autocrine or paracrine of Lgi1 was not prevented in our experiments. The mice with the specific deletion of Lgi1 in OL lineage cells will be a better model for a clear conclusion.

The secreted protein Lgi1 can interact with receptors such as ADAM22/23/11 (Kegel et al., 2014) and Nogo receptor 1 (NgR1; Thomas et al., 2010). However, none of these receptors is expressed in OLs. Leucine rich repeat and Immunoglobin-like domain-containing protein 1 (LINGO-1) is a transmembrane protein expressed in OLs and negatively regulates OPC differentiation and myelination (Mi et al., 2005; Jepson et al., 2012). Both Lgi1 and LINGO-1 are LRR-containing proteins, which might enable them to heterodimerize with each other. Alternatively, Lgi1 and LINGO-1 may compete with each other for binding to NgR1 and this competition may play an indirect role in CNS myelination (Figure 7D, red lines). The potential interaction between Lgi1 and LINGO-1 remains to be determined using transgenic mice with a mutation disrupting their association.

Balanced mTORC1 activity is required for CNS myelination. On one hand, mTORC1 activity is required for myelination (Flores et al., 2008) and *in vitro* or *in vivo* inhibition of mTOR signaling in OLs displays impaired OL development and hypomyelination in the CNS (Narayanan et al., 2009; Tyler et al., 2009; Zou et al., 2011, 2014; Guardiola-Diaz et al., 2012; Bercury et al., 2014; Wahl et al., 2014). On the other hand, overactivation of mTORC1 or TSC1 mutation causes hypomyelination in part by downregulating Akt signaling and lipogenic pathways as well as enhancing stress responses (Lebrun-Julien et al., 2014; Jiang et al., 2016). In the present work, we found that Lgi1 deficiency decreases the expression of TSC1 and activates mTORC1 in OLs, and *Lgi1*^−/−^ mice showed impaired lipogenic biosynthesis and loose myelin layers. These phenotypes corroborate with the importance of balanced mTORC1 activity in CNS myelination (Lebrun-Julien et al., 2014; Jiang et al., 2016). The finding that TSC1 expression was downregulated in *Lgi1*^−/−^ mice is unexpected and the function relationship between Lgi1 and TSC1 remains to be determined. Intriguingly, TSC1 mutant mice display a much reduced myelination (Meikle et al., 2007), in consistent with present study. The mutations in both Lgi1 and TSC1 lead to epileptogenesis (Meikle et al., 2007). The loss-of-function mutation of TSC1 in the cortex causes defective neuronal development at postnatal stages (Meikle et al., 2007; Normand et al., 2013; Tee et al., 2016). The ablation of Lgi1 also causes neuronal dyslamination in the cortex (Silva et al., 2015) and reduces the proliferation of granule precursor cells in embryonic CB (Xie et al., 2015). These observations suggest a potential correlation of Lgi1 and TSC1 functions not only in OPC differentiation but also myelin maintenance. In addition, it is possible that TSC2 is also involved in Lgi1-controlled myelination, because TSC2 deletion causes the hypomyelination in the brain (Carson et al., 2015).

Previous works show that Lgi1 inhibits the proliferation and causes the apoptosis of neuroblastoma cells (Kunapuli et al., 2003; Gabellini et al., 2006), which might be mediated by altered AKT and ERK (extracellular-signal-regulated kinase) signaling (Kunapuli et al., 2003; Sirerol-Piquer et al., 2006) However, the roles of Lgi1 in oncogenesis still remain quite unclear. We found that Lgi1 is critical for the maintenance of myelin in the brain, suggesting that Lgi1 loss may contribute to glioma-related dysmyelination. Future study of Lgi1 functions would need to define its biological functions in gliomagenesis and its therapeutic potential.

## Conclusion

Epileptic gene Lgi1 is necessary and sufficient for OPC differentiation and myelination, which may be associated with the modulation on the activity of TSC1-mTORC1 signaling.

## Author Contributions

Y-JX, YW and LiangZ designed the research. Y-JX, LinZ, N-WJ, SC, C-YS and X-TW performed the research. X-YL provided unpublished tools and techniques. Y-JX, LinZ and LiangZ analyzed the data. Y-JX, LinZ, YW, YS and LiangZ wrote the article. All authors read and approved the final manuscript.

## Conflict of Interest Statement

The authors declare that the research was conducted in the absence of any commercial or financial relationships that could be construed as a potential conflict of interest.
